# The reference genome of the Asian Elephant (*Elephas maximus*): a foundation for conservation and genomic research

**DOI:** 10.1186/s12864-026-12821-9

**Published:** 2026-04-13

**Authors:** Diego De Panis, Larissa S. Arantes, Tom Brown, Elisa Somenzi, Gudrun Wibbelt, Jennifer Ballaco, Jacquelyn Mountcastle, Nadolina Brajuka, Vinita S. Joardar, Olivier Fedrigo, Françoise Thibaud-Nissen, Oliver A. Ryder, Erich Jarvis, Virpi Lummaa, Camila J. Mazzoni

**Affiliations:** 1 Department of Evolutionary Genetics, Leibniz Institute for Zoo- and Wildlife Research (IZW), Berlin, Germany; 2 Berlin Center for Genomics in Biodiversity Research (BeGenDiv), Berlin, Germany; 3 Department of Biology, University of Turku, Turku, Finland; 4 Department of Wildlife Diseases, Leibniz Institute for Zoo- and Wildlife Research (IZW), Berlin, Germany; 5 Vertebrate Genome Laboratory, The Rockefeller University, New York, NY USA; 6 National Center for Biotechnology Information, National Library of Medicine, National Institutes of Health, Bethesda, MD 20894 USA; 7 Colossal Biosciences, Dallas, TX USA; 8 Conservation Science and Wildlife Health, San Diego Zoo Wildlife Alliance, Escondido, CA 92027 USA

**Keywords:** *Elephas maximus*, Asian elephant, Genome Assembly, Genome Annotation, Heterozygosity

## Abstract

**Background:**

The Asian elephant (*Elephas maximus*), a keystone species with both ecological and cultural significance, is highly endangered and has disappeared from 95% of its historical range. In this study, we present a chromosome-level assembly and an annotation of the Asian elephant genome, providing a foundational resource for population genomics, conservation biology and evolutionary research.

**Results:**

The primary genome assembly spans 190 contigs, with an N50 of 87,987,108 bp and is scaffolded into 64 sequences, with an N50 of 127,432,672 bp. We also present two haplotype-resolved assemblies with contig N50s of 75,101,715 bp and 88,213,608 bp. The genome assemblies and annotated protein-coding models in the primary assembly are highly complete, with 98.2%, 98.2%, and 96.0% BUSCO single-copy orthologs identified in the primary and two haplotype genome assemblies, respectively, and 98.8% recovered in the protein-coding annotation. We showcase how this reference genome enables insights into functional and evolutionary genomics, including the transposable element landscape, demographic history, a comparison against an individual sequenced from another population, as well as an investigation into genomic regions with increased levels of heterozygosity that colocalise with multi-copy gene families associated with immune and sensory-responses.

**Conclusion:**

The development of a high-quality genome assembly and annotation for *E. maximus* gives researchers a valuable resource to help understand the evolutionary history of this iconic species as well as guide conservation efforts. Here we have shown that highly contiguous, complete and accurate chromosome sequences help uncover regions with increased levels of homozygosity, indicative of inbreeding, and areas of increased heterozygosity, enriched for genes key to the immune response and other sensory mechanisms.

**Supplementary Information:**

The online version contains supplementary material available at 10.1186/s12864-026-12821-9.

## Background

Human-induced environmental changes are driving a rapid and unprecedented loss of biodiversity. The remaining wildlife is increasingly confined to small and fragmented populations that are particularly prone to present low genetic diversity and elevated inbreeding levels, which may reduce their adaptive potential and fitness [1]. A powerful emerging tool available for conservation biologists is the use of Genomics methods to understand factors influencing the persistence of endangered species [2]. In particular, reference genomes - highly contiguous and accurate genome assemblies - have become valuable resources for quickly assessing genomic diversity and architecture, ultimately aiding in species conservation [3]. These genomic resources have facilitated the understanding of unique species adaptations [4], the identification of the genetic basis of phenotypic traits [5] and wildlife diseases [6], the resolution of phylogenetic relationships [7] and the development of informed management strategies [8].

The Asian elephant (*Elephas maximus*) is one of the most iconic species on Earth, yet it is highly endangered, having disappeared from 95% of its historical range [9]. The remaining populations are experiencing rapid declines due to poaching, habitat loss, and increasing human-elephant conflicts [10]. Additionally, approximately one-quarter of the world’s Asian elephants now live in captivity, where they are used in tourism, temples, and the timber industry [11]. Beyond their ecological and cultural significance, Asian elephants exhibit complex social structures and possess remarkable biological traits. As long-lived mammals, they have developed robust disease defense mechanisms, including exceptional cancer resistance, which is linked to their multiple copies of tumor-suppressor genes [12]. Understanding these unique adaptations at the genomic level can provide valuable insights into longevity, disease resistance, and mammalian evolution.

A high-quality reference genome serves as a fundamental resource for addressing key questions in biology, disease, and conservation of the Asian elephant. By providing insights into the genetic basis of phenotypic traits, identifying signatures of selection, and uncovering adaptive genetic variation [13], a reference genome enables more effective conservation strategies. Additionally, it offers a higher-resolution view of chromosomal organization, facilitating comparative genomic and evolutionary analyses that contribute to our understanding of Afrotherian evolution.

In this work, we present and analyze a high-quality reference genome and annotation for the Asian elephant, which have been available since 2022 and have been in use by researchers from multiple research areas. For instance, it has been instrumental in identifying sex chromosomes in dugongs [14] and has facilitated comparative genomic analyses in Sirenians, shedding light on evolutionary relationships within this group [15]. Additionally, it has served as a benchmark for methodological advancements, including studies investigating the impact of reference bias in genomic analyses [16].

To demonstrate the quality of, and further broaden the use of this reference genome, we generated new haplotype-phased assemblies and compared them to another chromosome-level assembly from a specimen of different geographical origin [17]. We also analysed the composition and variability of both chromosome-level genomes to shed light on the Asian elephant’s transposable element landscape, underlying levels and patterns of heterozygosity, and demographic history. In addition, we identified genomic regions with increased levels of heterozygosity that are enriched for multi-copy gene families associated with the immune response and sensory activities, such as olfactory receptors.

## Methods

### Samples

For DNA sequencing, skin tissue and fibroblast cells were taken from the San Diego Zoo Wildlife Alliance’s Frozen Zoo^®^, part of the Wildlife Biodiversity Bank at the San Diego Zoo Wildlife Alliance. The biobanked individual was a 50 years old captive male from San Diego Zoo, California (SB-218, and GAN # 26734787; ZIMS/Species 360). Skin tissue and fibroblast cells were taken under IACUC protocol #15–017 for opportunistic collections taken during veterinary procedures, with the latter cultured for further research.

Five tissues (lung, thyroid, lymph node, salivary gland and ovary) from three Zoo individuals were taken from the pathology collection of the *Genome Resource Bank ARCHE*, Leibniz Institute for Zoo and Wildlife Research, Berlin for RNA sequencing. All specimens were taken post-mortem during pathology investigations into the cause of death. Tissue samples were removed at the time of necropsy, transported at + 4° C and stored at -80° C (445, 466) or snap-frozen in liquid nitrogen (291).

### Sample processing and sequencing

We isolated high molecular weight DNA from fibroblast cells provided by the Frozen Zoo. We used a Bionano SP Blood and Cell Culture DNA Isolation Kit (Bionano PN 80042) following the Frozen Cell Pellet DNA Isolation Protocol v2. We assessed DNA fragment size via pulsed field gel electrophoresis (Pippin Pulse, SAGE Science, Beverly, MA) and used a Qubit 3 fluorometer (Invitrogen Qubit dsDNA Broad Range Assay cat no. Q32850) for quantification.

For Bionano Optical Mapping, we labelled 750 ng of high molecular weight DNA with direct labeling enzyme (DLE1) following the Bionano Prep Direct Label and Stain (DLS) protocol (document number 30206) and imaged on a Bionano on a Bionano Saphyr instrument.

For PacBio sequencing, we sheared 15 µg of DNA to an insert size of 15 kb – 20 kb using a Megaruptor 3 (Diagenode, Denville, NJ, USA) and prepared a PacBio library with 10 µg of sheared DNA and the SMRTbell Express Template Prep Kit 2.0 (PacBio PN 100-938-900). We removed fragments under 5 kb from the library using Ampure PB beads (PacBio PN 100-265-900). We sequenced the library on a Sequel IIe instrument with 8 M SMRT cells, 30-hour movies time, 2-hour pre-extensive, and Sequencing Plate 2.0 (PN 101-820-200), generating 35x HiFi data.

We sent an aliquot of cells to the Arima Genomics sequencing facility (Arima Genomics, Carlsbad, CA, USA) to generate 70 X genome coverage Hi-C sequencing data using the Arima-HiC 2.0 kit.

We extracted RNA from five tissue samples using the RNeasy Mini Kit (QIAGEN) following the manufacturer’s protocol. We quantified RNA concentration using Qubit (Invitrogen), and assessed the quality by measuring the RNA Integrity Number (RIN) with an Agilent Fragment Analyzer. Only samples with a minimum RIN of 6 were included. We prepared RNAseq libraries using the TruSeq Stranded Total RNA Library Prep Kit (Illumina) and sequenced the libraries on a single lane of the NovaSeq 6000 SP platform with paired-end 150 bp reads at the Max Delbrück Center for Molecular Medicine in the Helmholtz Association.

### Assembly and functional annotation

We assembled the genome following the Vertebrate Genomes Project’s 2.0 pipeline [18] using 51x coverage PacBio HiFi reads, 300x coverage BioNano optical maps and 70x coverage Illumina reads from a Chromosomal Conformation Capture (Hi-C) library. After the initial assembly generated using only the PacBio HiFi reads as input to hifiasm (v0.16.1) [19] to create a set of primary “pseudo-haplotype” contigs, we generated two assemblies using the PacBio HiFi and Illumina Hi-C reads together as input to hifiasm (v0.19.5) to create two sets of haplotype-phased contigs following updates to the recommendations for submission of diploid assemblies by the Earth Biogenome Project https://www.earthbiogenome.org/report-on-assembly-standards. We then used purge-dups (v1.2.6) [20] to remove any haplotypic duplicate contigs present in each assembly. To initially scaffold into chromosomes, we used the Bionano optical maps and Bionano Solve’s hybrid-scaffold module (v3.3). We then mapped the Hi-C reads to the initial scaffolds using bwa-mem [21] and filtered the reads for mapping quality and PCR duplicates using picard, following the VGP’s Arima Hi-C mapping pipeline: https://github.com/VGP/vgp-assembly/blob/master/pipeline/salsa/arima_mapping_pipeline.sh.

We performed the final automated scaffolding with salsa2 (v2.3) [22] for the primary pseudo-haplotype assembly and with yahs (v1.2a.1) [23] for the haplotype-phased assemblies. We then performed a manual curation of the assembled scaffolds following the GRIT rapid-curation pipeline [24] to manually join any sequences from the same chromosome that were missed by the automated scaffolding tool and correct any false-joins or assembly errors. Finally, to assemble the mitochondrial sequence, we used oatk (v1.0) [25] using all adapter-trimmed PacBio HiFi reads as input and the Mammalia hmm models.

Functional elements of the primary genome, including protein-coding elements as well as non-coding genes and pseudogenes were annotated by the RefSeq team using The NCBI Eukaryotic Genome Annotation Pipeline, as described in Rhie et al. [26]. A total of 1.8 billion RNA-Seq reads from six tissues (lung, thyroid, scapula lymph node, salivary gland, ovary in SRP377913 and white blood cells in SRP065915), and all 65,292 RefSeq human proteins available on July 26, 2022 were used to inform gene calling.

### Gene family classification

To identify the major multi-copy gene family categories and positions in the reference genomes, we extracted the longest isoform for each gene using the AGAT toolkit (v1.4.2) [27], specifically using the agat_sp_keep_longest_isoform.pl command. We then obtained the corresponding protein sequences using the agat_sp_extract_sequences.pl command. These protein sequences were subsequently input into InterProScan (InterPro v5.63) [28, 29], utilizing the Pfam, PRINTS, SUPERFAMILY, PANTHER, Gene3D, FunFam, and SMART databases for comprehensive functional annotation. For genes belonging to multi-copy gene families, we classified them into specific categories, including “Major Histocompatibility Complex”, “Immunology-related”, “Olfactory Receptor”, and “Zinc-Finger”.

### Repeat masking

To mask repetitive regions of the genome, we first generated a *de-novo* repeat model library using RepeatModeler (v2.0.5) [30] and additional argument -LTRStruct using a combined assembly file of the primary and two haplotype-phased assemblies combined. The *de novo* repeats were combined with the ancestral repeat models for *E. maximus* in the curated Dfam library [31], which we obtained using the command “famdb.py families –format fasta_acc -ad –curated ‘Elephas maximus’ and the Dfam 3.8 library. The repeat models were first classified using RepeatClassifier (v2.0.5) and those that were listed as “Unknown” were then classified using DeepTE (commit babd65e) [32] using the Metazoa models. The combined, classified repeat models were then used to mask the repetitive regions of each genome using RepeatMasker (v4.1.6) [33] using the “ncbi” engine and arguments -xsmall -a. The kimura distance for each repeat was then calculated using the calcDivergenceFromAlign.pl script within RepeatMasker. The same analysis was performed on the primary assembly from Shi et al. [17] to allow for proper comparison (Fig. S1).

### Synteny analysis of chromosome-level assemblies

To investigate genome synteny between the genomes of *E. maximus* individuals from India and China, we identified syntenic regions of the genome based on predicted gene locations. For each assembly (primary, haplotype 1 & 2 and the primary assembly from [17]) we predicted gene models with Helixer (v0.3.0) using the Vertebrata model [34] and then detected orthologous protein sequences and linkage groups using the odp script “odp_nway_rbh” (v0.3.3) [35]. To generate a broad overview of syntenic regions between genome assemblies, we used the D-GENIES platform [36] to find regions mapping between chromosomes based on the output of minimap2 [37].

### Genomic diversity distribution

To investigate patterns of genetic diversity in the Asian Elephant genomes from India and China, we performed variant calling using the jATG pipeline (https://github.com/diegomics/jATG/tree/devel*).* First, PacBio HiFi reads were mapped to the primary genome assembly using minimap2 (v2.26) [37]. After mapping, we used MarkDuplicates from GATK (v4.6) [38] to remove PCR duplicates from the BAM files and then called variant sites using GATK HaplotypeCaller and GenotypeGVCF. We performed four variant calling using each genome HiFi data against its own assemblies, both primary and haplotype 1 assemblies (i.e. PacBio HiFi reads from BioProject PRJNA1008098 mapped to the assembly GCF_024166365.1 for the Indian individual and from project PRJCA018778 to the assembly, GCA_033060105.1 for the Chinese individual).

Following variant calling, we filtered the resulting gVCF using BCFtools [39]. We restricted the dataset to high-quality biallelic SNPs by excluding non-SNP variants and multiallelic sites. We applied strict quality control, filtering positions with low mapping quality, or where depth or genotype quality information was missing. We also applied depth thresholds, excluding sites with depth below 8 or greater than twice the mean coverage. To minimize genotyping errors, we removed sites with missing genotypes despite adequate coverage and applied allele balance filters: excluding heterozygotes deviating from the expected ratio and homozygous alternate calls with excessive reference support. Finally, we removed technical artifacts, including gVCF reference blocks and positions with undefined reference bases. We ran the pipeline only on the 27 autosomes for downstream analyses. After filtering, we converted all remaining genotypes to missing data, generating a base-pair resolution gVCF file. Positions located in masked regions were excluded for demographic analysis.

We used the filtered gVCF file for genome-wide heterozygosity estimation and runs of homozygosity (RoH) analysis using Darwindow [40]. We assessed heterozygosity via a sliding-window approach with non-overlapping 20 kb windows. We identified RoH segments based on a heterozygosity threshold calculated from the genome-wide mean heterozygosity, with genomic windows classified as a low-heterozygosity region if its heterozygosity fell below one-fifth of the mean heterozygosity (parameter hethres_vec = 0.04). We defined RoH segments as contiguous regions of at least 500 kb. Additionally, we allowed windows to contain a maximum of 20% missing data. We calculated the inbreeding coefficient (FRoH) as the proportion of the genome classified as RoH, providing insights into levels of inbreeding and historical population structure.

We then plotted the distribution of genomic diversity along the chromosomes alongside multi-copy gene families to visually identify any co-localized patterns. We tested the enrichment of these multi-copy gene families against all other protein-coding genes annotated via Fisher’s exact test, followed by Benjamini-Hochberg correction of p-values to account for multiple testing.

### Demographic analysis

We inferred the demographic history of the Asian elephant using the Pairwise Sequentially Markovian Coalescent (PSMC) model [41] for both chromosome-level assemblies of individuals from India and China. First, we extracted the consensus sequence from the filtered gVCF files generated as described above using BCFtools [39]. The resulting consensus fasta file was then converted into the PSMC input format using the fq2psmcfa tool. PSMC was run with default parameters: -N25 -t15 -r5 -p “4 + 25*2 + 4+6”, with scaling based on a mutation rate of 1.3 × 10⁻⁸ mutations per site per generation and a generation time of 31 years, following Palkopoulou et al. [42]. We performed 10 bootstrap replicates by randomly sampling with replacement from the consensus sequence.

## Results

### Genome assembly

**Fig. 1:**
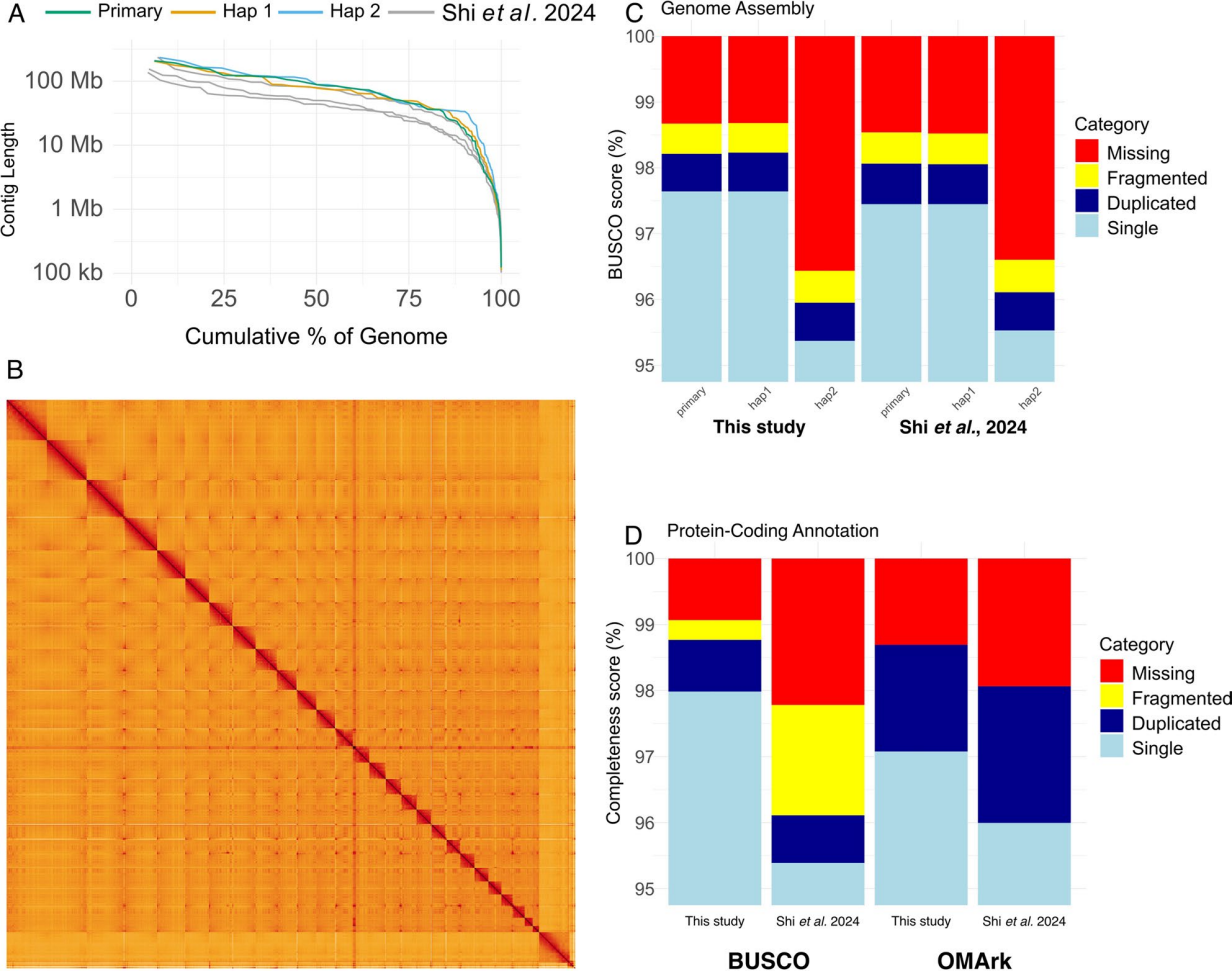
**A** Nx plot showing the lengths of contigs in the primary and two haplotype-resolved assemblies (colored) and available chromosome-level assemblies on NCBI (grey) for *E. maximus*. Contigs are ordered by length (y-axis) showing the composition of longest contigs for the cumulative proportion of the genome (x-axis). **B** Hi-C plot showing the interactions in 3-dimensional space between each area of the genome in the primary assembly. The diagonal shows self-interactions and red squares assembled chromosomes. **C** BUSCO gene completeness using eutheria_odb10 database for the primary and haplotype-resolved assemblies in this study and from Shi et al. [17] (**D**) BUSCO and OMArk completeness scores for the annotated protein sequences from the primary assemblies from this study and Shi et al. [17]. Note the y-axes for panels (**C**) and (**D**) begin at 95%

We assembled the primary assembly and two haplotype-phased assemblies into highly contiguous sequences, with contig N50 values of 88 Mb, 78 Mb and 88 Mb and N90 values of 17 Mb, 19 Mb and 34 Mb respectively for each assembly (Table S1), which are each more contiguous and complete than the available chromosome-level genome assemblies on NCBI (Fig. 1A) [17]. The genome assemblies are also highly complete, with over 98% complete BUSCO genes from the eutheria lineage identified in our primary and first haplotype assembly and over 95% in the second haplotype assembly containing the Y chromosomes and not X (Fig. 1C and Tables S2-4). The assemblies are scaffolded into chromosome molecules, with 99.1%, 97.7% and 98.8% of the assemblies contained within the chromosomal molecules, respectively (Fig. 1B). Syntenic maps of the primary genome assembly to the GRCh38 assembly for *Homo sapiens* is available via NCBI’s Comparative Genome Viewer (CGV) [43] at the following location: https://www.ncbi.nlm.nih.gov/cgv/browse/GCF_024166365.1/GCF_000001405.40/64955/99487.

### Protein-coding annotation

The gene annotation of the sequences in the primary assembly contains 21,809 protein sequences and over 5,000 non-coding genes (Table 1). The annotation is also highly complete, with 98.8% BUSCO and 98.7% OMArk orthologs identified in the annotated protein sequences, with a consistency score of 98.31% as identified by OMArk (Fig. 1D; Table 2). Furthermore Gene Ontology terms were assigned to predicted gene models and are available alongside the annotation files via the NCBI ftp: https://ftp.ncbi.nlm.nih.gov/genomes/all/GCF/024/166/365/GCF_024166365.1_mEleMax1_primary_haplotype/GCF_024166365.1-RS_2023_02_gene_ontology.gaf.gz.

**Table 1:** Statistics from the annotated gene models in the annotation of the primary genome assembly

Category	No. genes	No. transcripts	Mean gene length (bp)	No. single-exon genes	Mean exons per transcript
Protein-coding	21,809	53,692	60,529	3,285	12.6
lncRNA	2,045	3,543	37,080	0	4.3
snRNA	775	775	113	775	1
snoRNA	859	859	92	859	1
rRNA	95	95	1,229	95	1
tRNA	1,433	1,433	75	1,374	1

**Table 2:** Annotation completeness and consistency statistics evaluated via BUSCO and OMArk using Eutheria databases of conserved orthologs

	Complete	Singular	Duplicated	Fragmented	Missing
BUSCO	98.8% (11,226)	98.0% (11,137)	0.8% (89)	0.3% (34)	0.9% (106)
OMArk	98.69% (13,858)	97.08% (13,453)	1.62% (224)	-	1.31% (181)
	Consistent	Inconsistent	Contaminants	Unknown	
OMArk	98.31% (21,631)	0.82% (181)	0% (0)	0.86% (190)	

### Repeat-region annotation

**Fig. 2:**
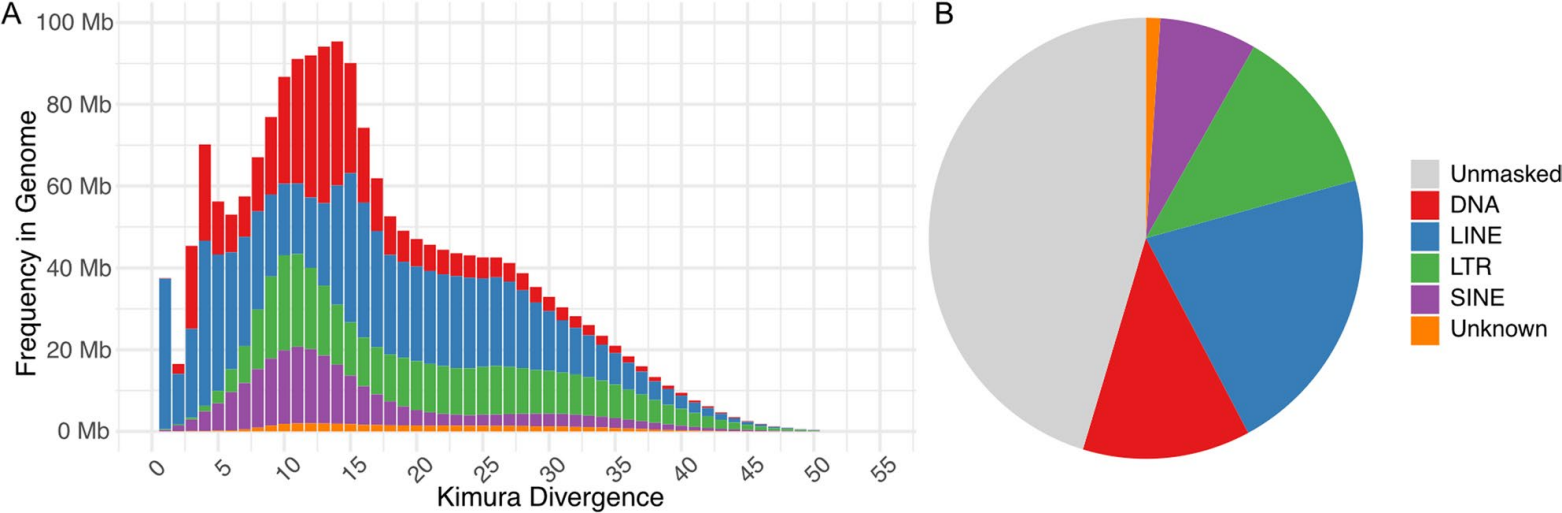
**A** Kimura divergence frequency of identified repeat elements in the primary genome assembly coloured by their category. **B** Distribution of repeat elements in the primary genome assembly coloured by their category

Of the 55% of the genome masked for repetitive and transposable elements (TE), the majority of the repetitive regions masked in the genome are made up of longer LINE elements (38%), LTR elements (22%) or DNA transposons (22%) (Figs. 2B, S1 and Table S5). Kimura distances were calculated for all types of TEs in order to estimate the age of transposable element activity in a genome. Low Kimura substitution levels indicate recent transposition events, while higher levels suggest older activity [44]. We found via Kimura divergence a recent burst of activity in all categories of repeats, with only LINE and LTR elements showing older activity of transposable elements (Fig. 2A).

### Patterns of genomic diversity and functional features

**Fig. 3:**
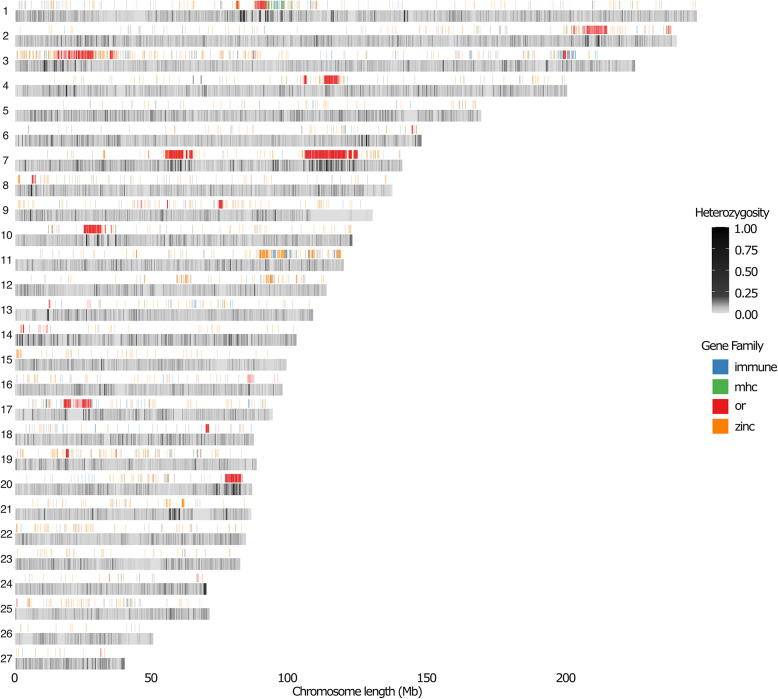
Genomic Heterozygosity and Multi-Copy Gene Family Distribution. This heatmap depicts Heterozygosity (He) across the 27 autosomes, with He values normalised using min–max scaling across the entire genome shown in non-overlapping 100 kb windows. The distribution of multi-copy gene families is overlaid, with each family color-coded according to its annotation

Our analysis of average heterozygosity across chromosomes revealed a non-uniform distribution, with increased heterozygosity concentrated in specific hotspot regions (Fig. 3). We then explored the association between genetic diversity and multi-copy gene families involved in immune response, olfactory receptors (ORs), major histocompatibility complex (MHC) and zinc fingers. We observed that OR genes were significantly clustered in regions of high heterozygosity (adjusted *p* < 10^− 22^), especially on chromosomes 1, 2, 3, 4, 7, 10, and 20, while MHC genes were concentrated in a genetic diversity hotspot on chromosome 1 (adjusted *p* < 10^− 19^). Zinc finger genes, on the other hand, were predominantly located in a region on chromosome 11, but did not show an association with increased heterozygosity (adjusted *p* = 0.9748). Similarly, immune-related genes were more evenly distributed across the genome without any clear link to increased heterozygosity (adjusted *p* = 0.7536).

**Fig. 4:**
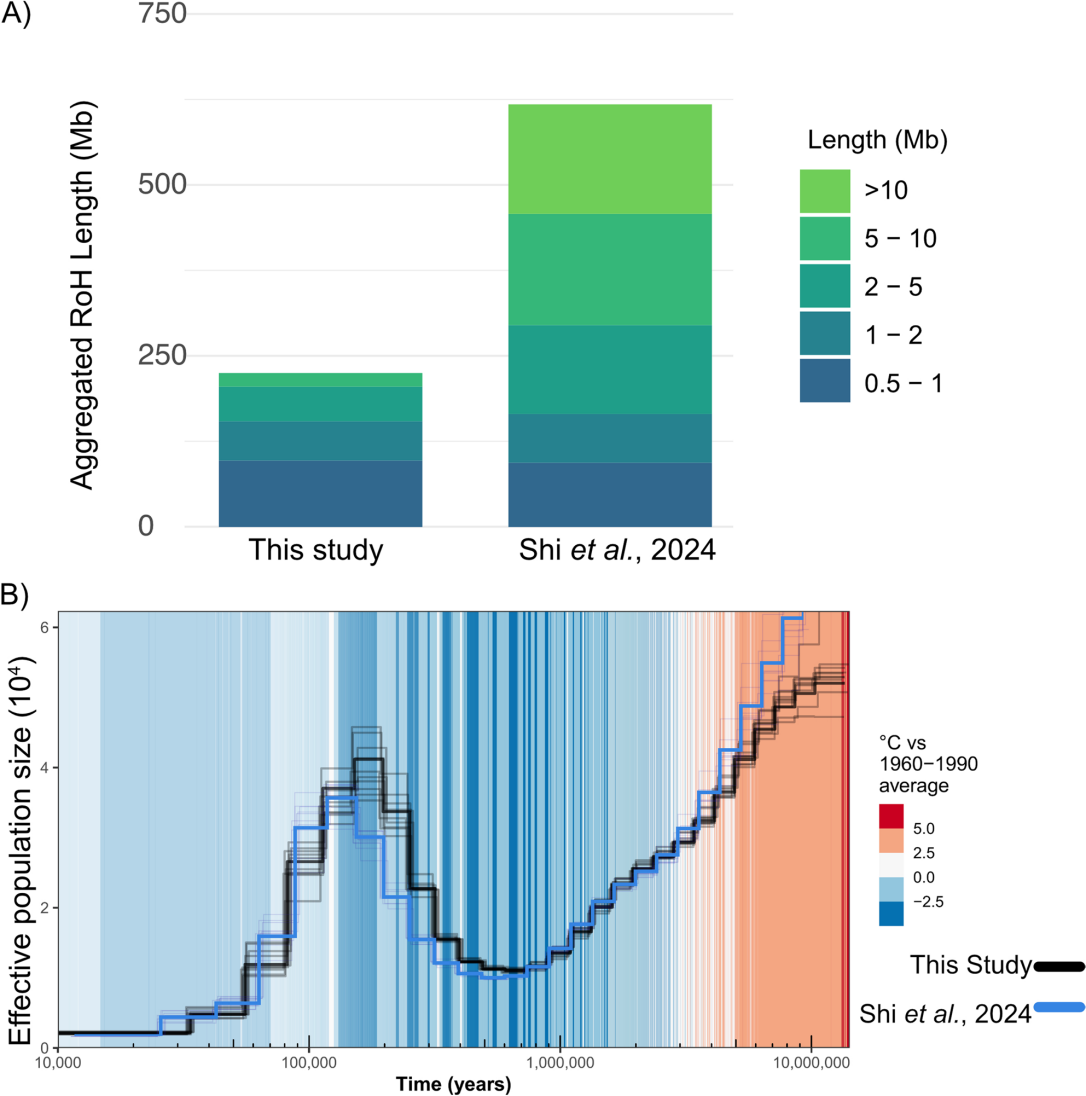
**A** Aggregated Runs of Homozygosity (RoH) categorized by segment length (in Mb) for sequenced individuals from India (left, this study) and China (right, Shi et al., 2024 [17]). Longer RoH suggests more recent common ancestry between an individual’s maternal and paternal lineages, whereas shorter RoH indicates older inbreeding events. **B** Demographic history of Asian elephants. Shown are the inferred histories based on the individuals sequenced from India (black, this study) and China (blue, Shi et al. [17]) Changes in effective population size over time were estimated using the PSMC model. Demographic trajectories were scaled by a per-generation mutation rate of 1.3 × 10⁻⁸ and a generation time of 31 years. The graph is overlaid with temperature fluctuations to highlight potential correlations between climate change and population dynamics. Bootstrap replicates are shown in lighter colors. The temperature scale represents temperature anomalies, i.e., the difference between reconstructed temperatures and the mean temperature of the 1960–1990 reference period. Positive values indicate temperatures warmer than the 1960–1990 average, and negative values indicate cooler conditions

Runs of Homozygosity (RoH) analysis allow us to identify genomic regions where both haplotypes are identical due to inheritance from a common ancestor, providing insights into individual inbreeding levels and population demographic history [45]. Our results indicate that the sequenced Asian elephant exhibits a low level of inbreeding, with only a few long RoH segments (Fig. 4A). This is contrasting with what we obtained for a recently published genome from a Chinese population [17], which displays a predominance of long RoH indicative of recent parental relatedness.

### Demographic analysis

The PSMC analysis for the Asian elephant shows a population decline starting around 10 million years ago (mya) during the Miocene. Subsequently, the Ne increases reaching a peak around 120 thousand years ago (kya) in the Last Interglacial, also known as the Eemian period (Fig. 4B). These time periods coincided with cooler glacial and warmer interglacial periods that were responsible for demographic changes in many species. In the Late Pleistocene, the Asian elephant population experienced a sharp decline. The inferred demographic history was also found to be consistent when using the genome assembly and WGS data from the individual from a population in China [17] (Fig. 4B).

### Genome-wide synteny

**Fig. 5:**
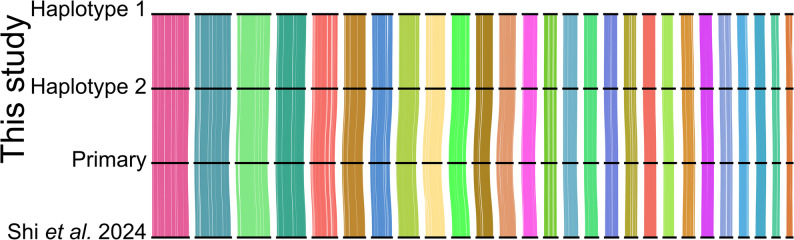
Locations of orthologous protein-coding genes identified between the autosomes of Elephas maximus genomes from individuals from India (top 3 genomes, this study) and China (bottom genome Shi et al. [17]). Horizontal black bars correspond to the chromosomes, ordered by numbering in the reference. Ribbons are coloured by inferred linkage groups and each vertical line represents a single reciprocal-best-hit between protein sequences in the respective genomes

Identification of orthologous protein sequences and their locations in the Indian and Chinese *E. maximus* individuals revealed large degrees of synteny, with all chromosomes showing strong collinearity and no rearrangement, including between the two haplotype-level assemblies generated (Fig. 5). The only exception was the Y chromosome, which appears to be mislabelled in the published genome available on GenBank (GCA_033060105.1). Following our comparisons, we found that the scaffold 30 in the Chinese individual genome assembly shows homology to the Y chromosome assembled for the Indian individual genome and published African elephant (*Loxodonta africana*) from the same study (GCA_033060095.1 (Fig. S2)).

## Discussion

Here we present a high-quality reference genome for the Asian Elephant, facilitating investigation of genome-level synteny, the transposable element landscape in *E. maximus*, the interplay between genetic variation and functional annotations, and the species’ demographic history. While overall there were no major rearrangements or large structural variations between the two haplotypes of the sequenced individual (Fig. 5), we still uncovered regions of higher heterozygosity in the genome, often clustering with the highly variable multi-copy gene families such as the major histocompatibility complex (MHC) or olfactory receptors (OR) (Fig. 3). The expanded repertoire of olfactory receptors has been highlighted previously in herbivores and shown to correlate with diet among mammals as a whole [46–48], suggesting that not only is the increased number in the genome as a whole important, but also the multiple copies found in both haplotypes for each individual.

Through investigation of the length of homozygous regions of the genome, we found less than 10% of the genome in runs of homozygosity (RoH) and the majority of the identified RoHs were short in length (< 1 Mb), indicating little evidence of recent inbreeding. The identified demographic history is similar to previously published results [17, 49] showing a population peak during the Eemian warming period before the population decline in the recent history of the species, which coincides with intensified human activities across various continents [50, 51]. This demographic history mirrors the patterns we obtained in a published Chinese population genome [17], confirming a shared evolutionary trajectory. However, recent ancestry profiles diverge significantly. Unlike the Shi et al. individual, which exhibits a signal of long RoH indicative of recent inbreeding, our individual retains a diverse, outbred background. This suggests our reference assembly could be used as a more representative proxy for the historical baseline of genetic diversity within the species.

The assembly of all sequences into highly contiguous chromosomal molecules facilitated such investigation, with high levels of confidence in the underlying sequence and structure of the assembled genome thanks to long contigs and highly accurate PacBio reads. A key aspect of achieving this high contiguity, including the improved resolution of complex sex chromosomes, was rigorous manual curation. This establishes the current assembly and its phased haplotypes as a robust reference, minimizing the bias often introduced by fixing deleterious alleles in highly homozygous samples.

## Conclusion

The generation of this high-quality assembly and annotation for the Asian Elephant for use by the scientific community has already assisted researchers in both evolutionary and conservation studies focusing on this enigmatic endangered species. Here, we have demonstrated both the quality and usefulness of such resources, highlighting how highly accurate, contiguous, chromosome-scale sequences allow us to investigate a number of features of the genetic backbone using a single resource. The increase in genetic diversity in regions of the genome harbouring genes driving with the immune and sensory responses as well as an understanding of the regions of the genome exhibiting larger regions of homozygosity, indicative of inbreeding within the population, help lay the foundation for others to determine markers within the genome key to supporting elephant populations and breeding in the future.

## Supplementary Information

Supplementary Material 1.

## Data Availability

The data described in this article can be freely and openly accessed via the International Nucleotide Sequence Database Collaboration (INSDC) via the following accessions: Umbrella Genome BioProject: PRJNA855931, RNA-seq BioProject: PRJNA844231, Genomic sequencing data BioProject: PRJNA1008098, Primary genome and annotation: GCF_024166365.1, Hap1/2 assemblies: JBNQWU000000000 and JBNQWV000000000. Scripts used to generate figures, tables and statistics including heterozygosity analysis, demographic history analysis, repeat landscape annotation and synteny analysis are available at the following gitlab repository: https:/git.imp.fu-berlin.de/begendiv/melemax_genome.
