# Supplementary material for: The reference genome of the Asian Elephant (Elephas maximus): a foundation for conservation and genomic research
Source: BMC Genomics. 2026 Apr 13;27:486. doi: 10.1186/s12864-026-12821-9 (PMC13185315; doi:10.1186/s12864-026-12821-9)
Supplement: Supplementary file 1 — Supplementary Material 1. [file 12864_2026_12821_MOESM1_ESM.docx]

Supplementary Material


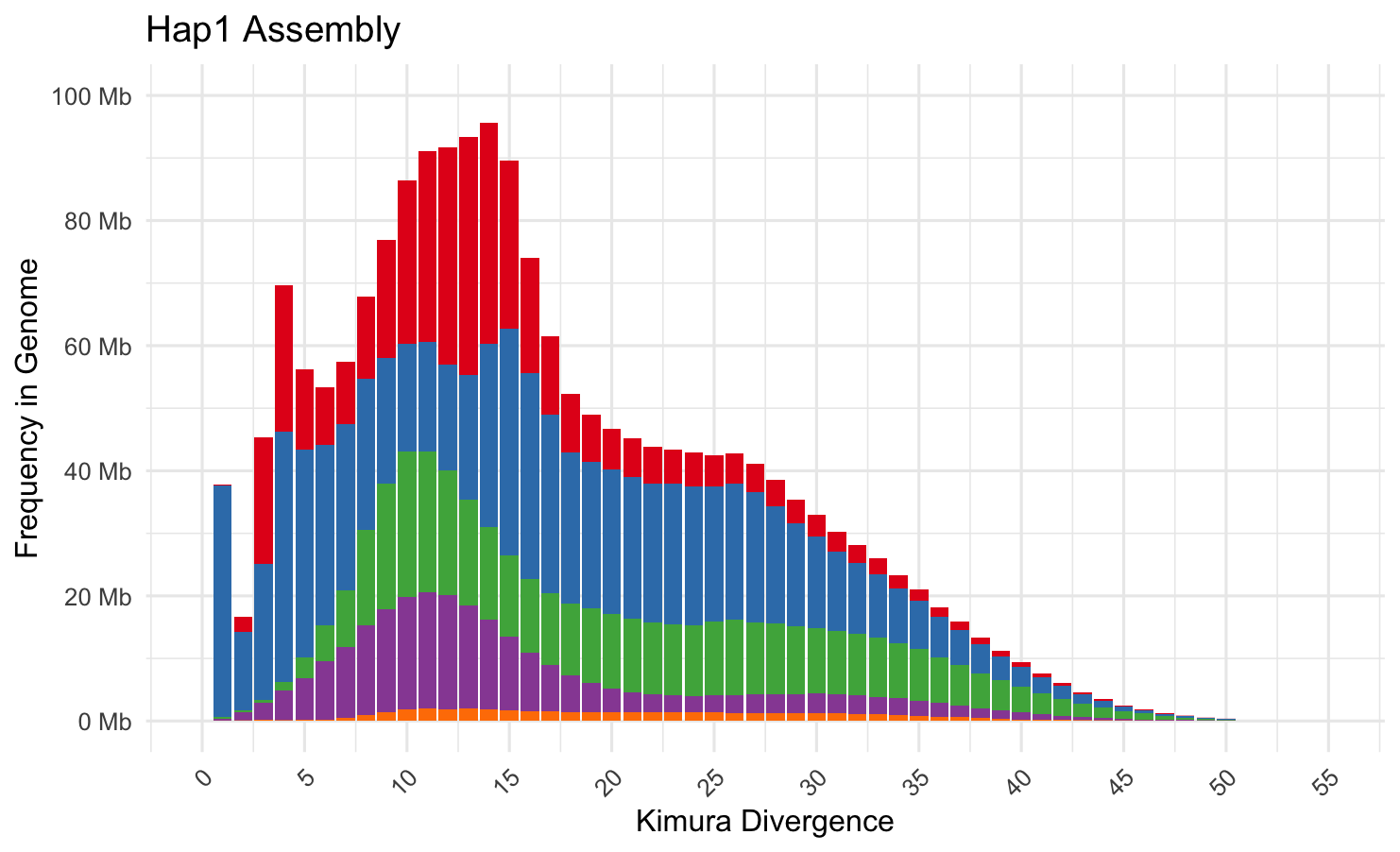

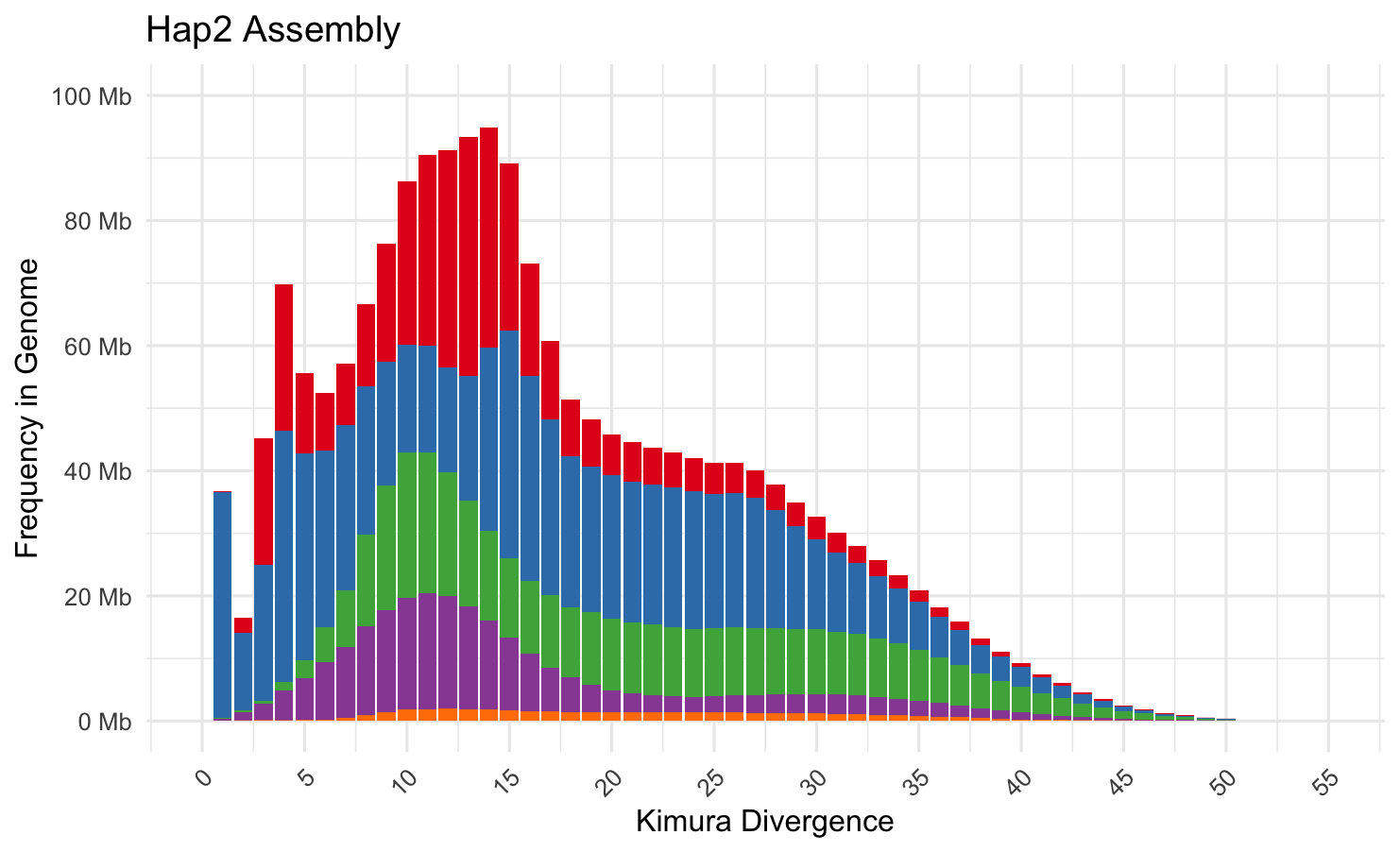

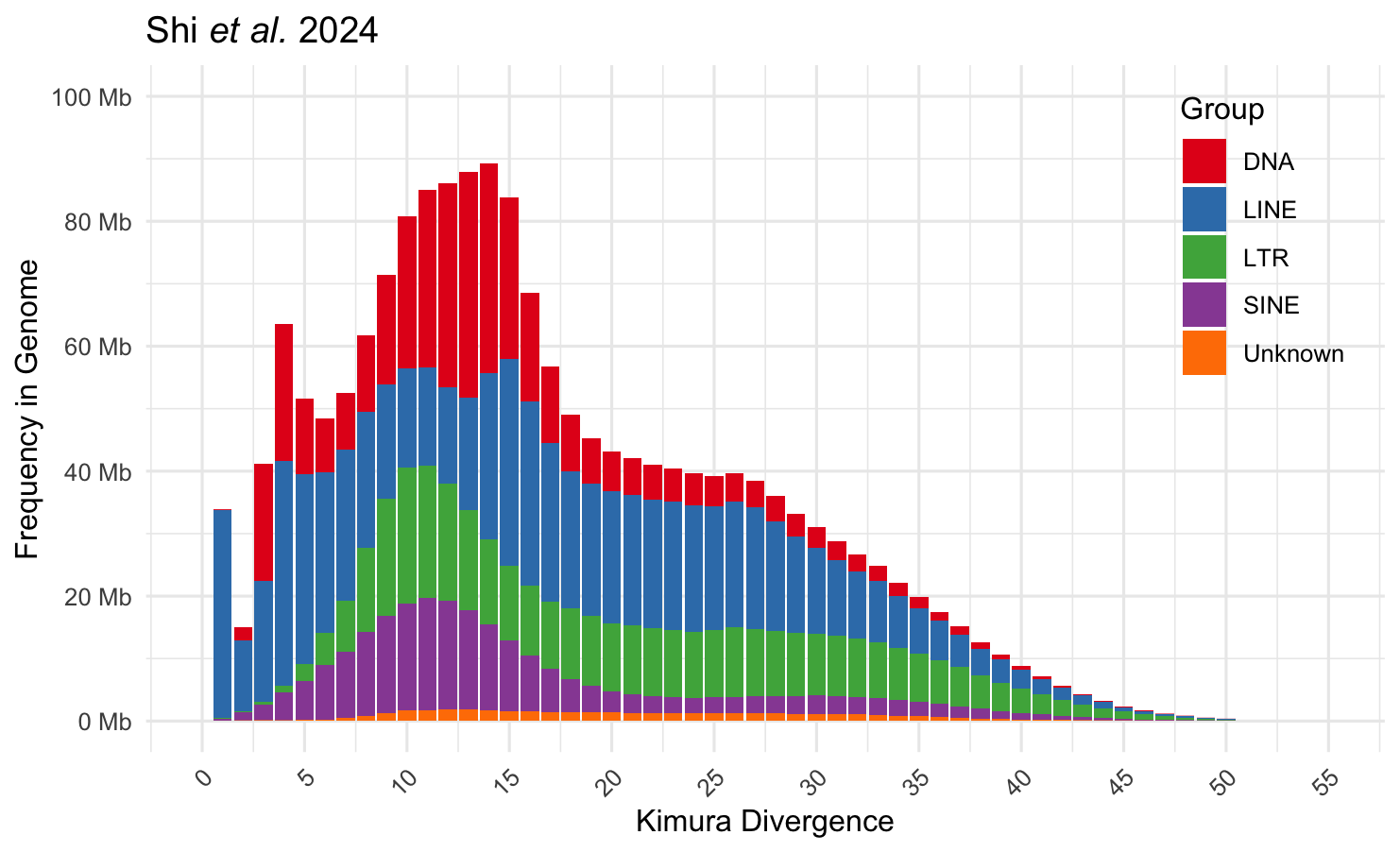

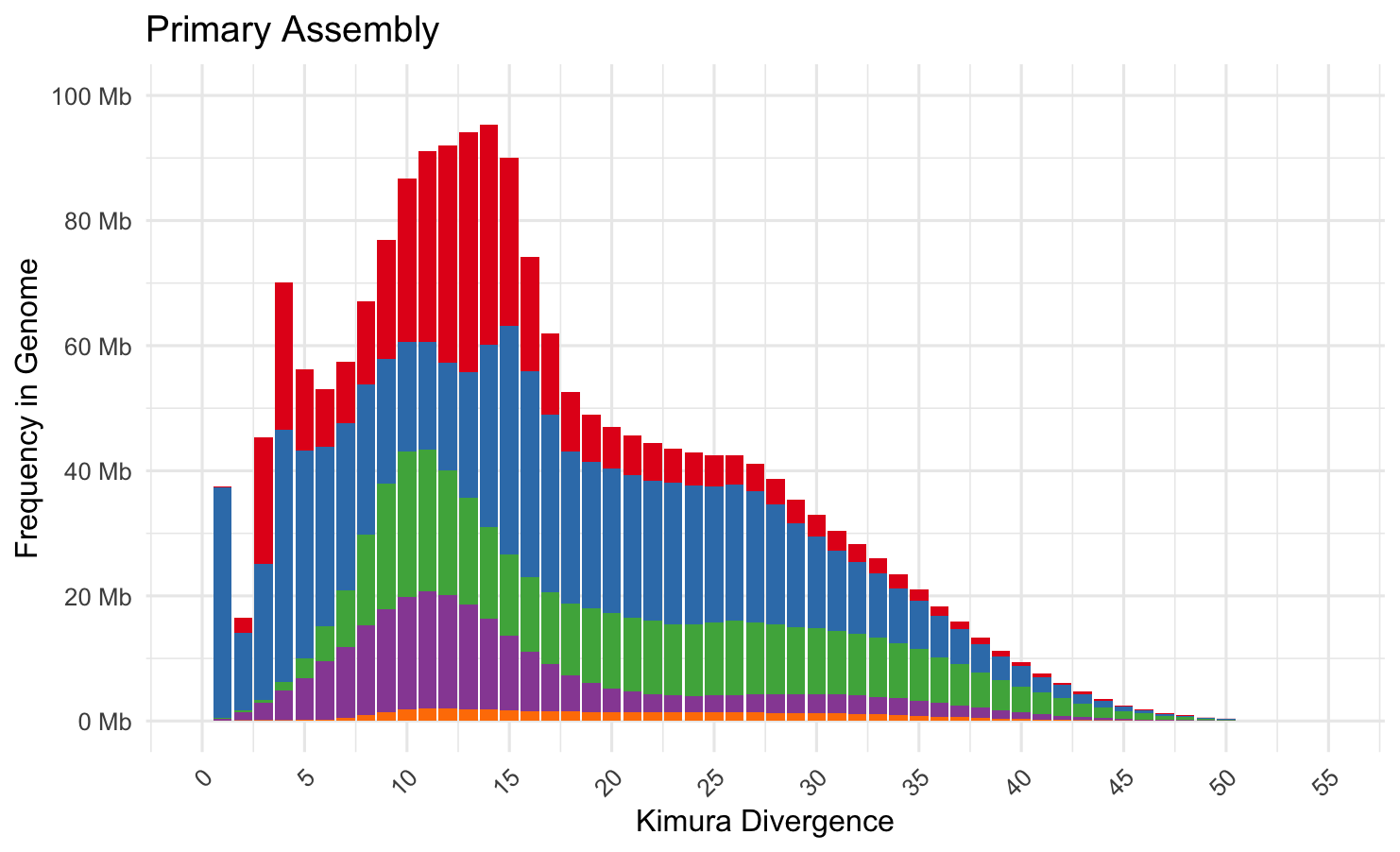


Figure S1: Repeat landscapes for the primary assemblies from this study (top-left), the sequenced individual from a Chinese population (top-right) and the hap1 and hap2 (lower-left and right) assemblies from this study.


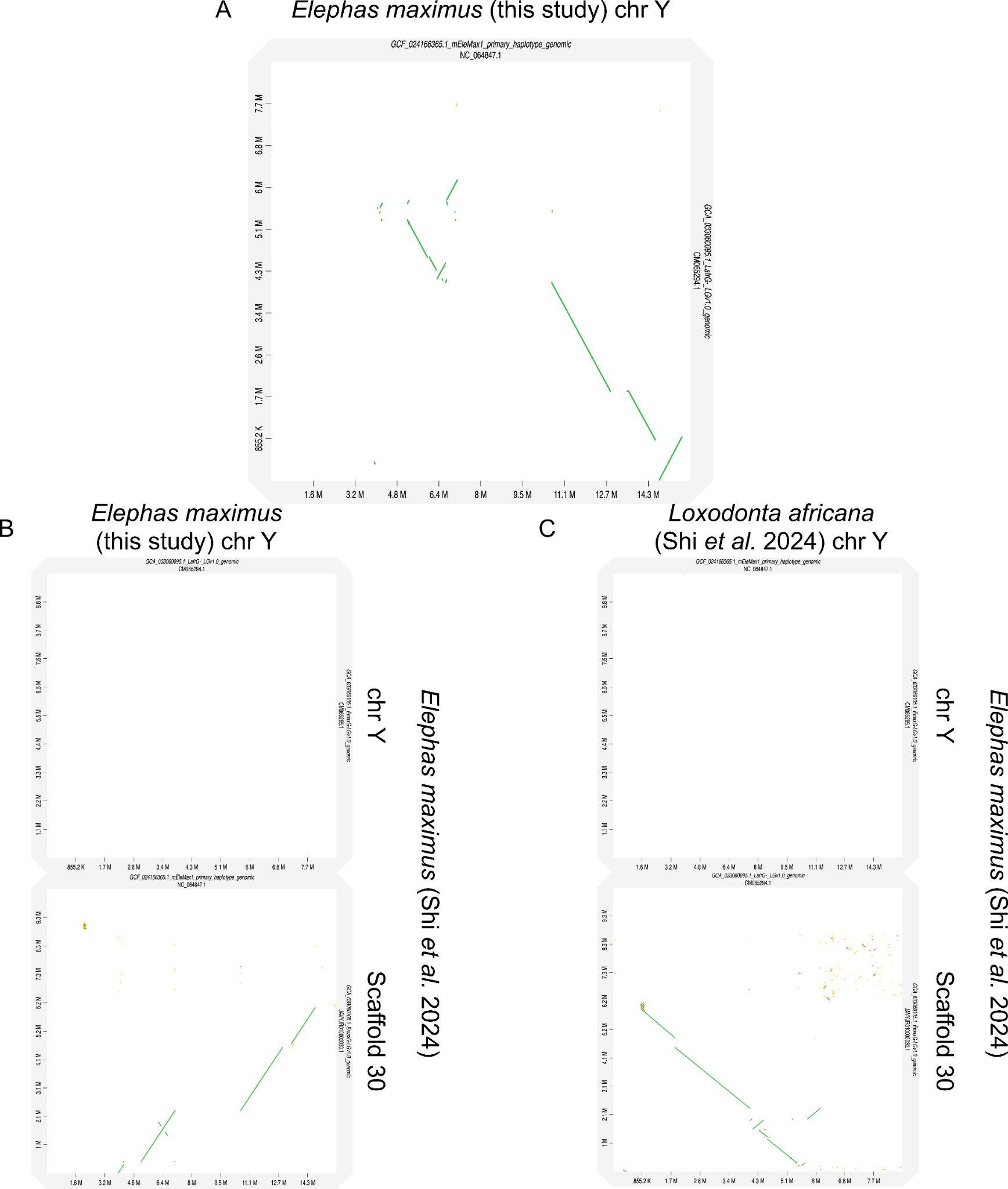


Figure S2: Dotplots showing identified syntenic regions of the labelled and inferred Y chromosomes between the assembly presented here (A and B horizontal axis), the Asian elephant from Shi *et al.* 2024 (B and C vertical axis) and the African elephant genome from the same study (A vertical axis and C horizontal axis). Shown are regions with >50% mapped regions as detected by minimap. Panels B and C show that scaffold 30 in the released Asian elephant genome shows synteny to the Y chromosomes from other elephant genomes.

|  | | | Size (Mb) | | | No. Scaffolds | | | Scaffold N50 (Mb) | | | Scaffold N90 (Mb) | | | No. Contigs | | | Contig N50 (Mb) | | | Contig N90 (Mb) | | | QV |
| --- | --- | --- | --- | --- | --- | --- | --- | --- | --- | --- | --- | --- | --- | --- | --- | --- | --- | --- | --- | --- | --- | --- | --- | --- |
| pri | | | 3,401 | | | 64 | | | 127 | | | 79.9 | | | 190 | | | 88.0 | | | 17.3 | | | 68.2 |
| hap1 | | | 3,343 | | | 104 | | | 125 | | | 73.3 | | | 178 | | | 77.7 | | | 19.3 | | | 69.2 |
| hap2 | | | 3,141 | | | 90 | | | 126 | | | 78.4 | | | 152 | | | 88.2 | | | 33.6 | | | 69.4 |

Table S1: Genome Assembly statistics for the primary (pri) and two haplotype-phased assemblies (hap1 and hap2). QV: Quality Value

| BUSCO metaeuk (n=11,366) | | | | | |
| --- | --- | --- | --- | --- | --- |
|  | Complete | Single | Duplicate | Fragmented | Missing |
| pri | 94.9% (10,777) | 94.1% (10,690) | 0.8% (87) | 0.9% (100) | 4.2% (489) |
| hap1 | 92.9% (10,559) | 92.1% (10,465) | 0.8% (94) | 1.1% (129) | 6.0% (678) |
| hap2 | 90.5% (10,285) | 89.8% (10,201) | 0.7% (84) | 1.2% (132) | 8.3% (949) |

Table S2: Benchmarking Universal Single Copy Ortholog (BUSCO) scores for the three genome assemblies using the metaeuk mapping tool

| BUSCO miniprot (n=11,366) | | | | | |
| --- | --- | --- | --- | --- | --- |
|  | Complete | Single | Duplicate | Fragmented | Missing |
| pri | 98.2%(11,163) | 97.6% (11,098) | 0.6% (65) | 0.5% (52) | 1.3% (151) |
| hap1 | 98.2% (11,165) | 97.6% (11,098) | 0.6% (67) | 0.5% (51) | 1.3% (150) |
| hap2 | 96.0% (10,906) | 95.4% (10,840) | 0.6% (66) | 0.5% (55) | 3.5% (405) |

Table S3: Benchmarking Universal Single Copy Ortholog (BUSCO) scores for the three genome assemblies using the miniprot mapping tool

| Compleasm (n=11,366) | | | | | |
| --- | --- | --- | --- | --- | --- |
|  | Complete | Single | Duplicate | Fragmented | Missing |
| pri | 99.75% (11,338) | 99.46% (11,305) | 0.29% (33) | 0.04% (4) | 0.21% (24) |
| hap1 | 99.46% (11,341) | 99.46% (11,305) | 0.32% (36) | 0.03% (3) | 0.19% (22) |
| hap2 | 97.40% (11,070) | 97.09% (11,035) | 0.31% (35) | 0.08% (9) | 2.53% (287) |

Table S4: Gene completeness statistics based on the compleasm tool of the primary (pri) and two haplotype-phased assemblies assemblies

| **Category** | **Primary** | **Hap1** | **Hap2** | **Shi *et al.* 2024** |
| --- | --- | --- | --- | --- |
| **SINE** | 242,481,291bp / 7.13% | 238,236,075bp / 7.29% | 229,244,131bp / 7.39% | 241,460,935bp / 7.15% |
| **Penelope** | 11,697 bp / 0.00% | 11,609bp / 0.00% | 11,308bp / 0.00% | 11,530bp / 0.00% |
| **LINE** | 718,504,894bp / 21.12% | 711,853,632bp / 21.79% | 654,373,368bp / 21.08% | 717,679,707bp / 21.25% |
| **LTR element** | 418,249,509bp / 12.3% | 401,178,388bp / 12.28% | 383,821,448bp / 12.37% | 417,783,847bp / 12.37% |
| **DNA transposon** | 418,937,416bp / 12.32% | 416,353,088bp / 12.7474% | 394,538,929bp / 12.71% | 417,513,955bp / 12.36% |
| **Rolling-circles** | 251,177bp / 0.01% | 250,956bp / 0.01% | 242,567bp / 0.01% | 250,544bp / 0.01% |
| **Unclassified** | 35,780,583bp / 1.03% | 35,471,762bp / 1.09% | 33,220,198bp / 1.07% | 35,695,857bp / 1.06% |
| **Total Interspersed Repeats** | **1,881,836,214bp / 55.33%** | **1,842,991,260bp / 56.41%** | **1,732,381,472bp / 55.82%** | **1,830,145,831bp / 54.18%** |
| **Small RNA** | 242,395,488bp / 7.13% | 238,150,838bp / 7.29% | 229,162,831bp / 7.38% | 241,375,902bp / 7.15% |
| **Simple Repeats** | 39,614,683bp / 1.16% | 33,519,921 / 1.03% | 30,849,310bp / 0.99% | 40,466,366bp / 1.20% |
| **Low Complexity** | 8,000,464bp / 0.24% | 6,111,340 / 0.19% | 6,075,724bp / 0.2% | 8,162,552bp / 0.24% |

Table S5: Proportion of the genome annotated as belonging to each family of repetitive elements.
